# Zinc oxide and copper oxide nanoparticles as a potential solution for controlling *Phytophthora infestans,* the late blight disease of potatoes

**DOI:** 10.1186/s11671-024-04040-6

**Published:** 2024-06-22

**Authors:** Amira A. AlHarethi, Qais Y. Abdullah, Hala J. AlJobory, AbdulRahman M. Anam, Ramadan A. Arafa, Khaled Y. Farroh

**Affiliations:** 1 Department of Biological Science, Faculty of Science, Sana’a University, Sana’a, Yemen; 2 Department of Pharmacology, Faculty of Medicine and Health Science, Sana’a University, Sana’a, Yemen; 3 Plant Pathology Research Institute, Agricultural Research Center, Giza, 12619 Egypt; 4 Nanotechnology and Advanced Materials Central Lab, Agricultural Research Center, Giza, Egypt

**Keywords:** *P. infestans*, Zinc oxide (ZnO) and Copper oxide (CuO) nanoparticles (NPs), Synthesis and characterization, In vitro, Greenhouse conditions, Antifungal activity

## Abstract

Late blight, caused by *Phytophthora infestans*, is a major potato disease globally, leading to significant economic losses of $6.7 billion. To address this issue, we evaluated the antifungal activity of ZnO and CuO nanoparticles (NPs) against *P. infestans* for the first time in laboratory and greenhouse conditions. Nanoparticles were synthesized via a chemical precipitation method and characterized using various techniques. The XRD results revealed that the synthesized ZnO nanoparticles had a pure hexagonal wurtzite crystalline structure, whereas the CuO NPs had a monoclinic crystalline structure. TEM images confirmed the synthesis of quasi-spherical nanoparticles with an average size of 11.5 nm for ZnO NPs and 24.5 nm for CuO NPs. The UV–Vis Spectral Report showed peaks corresponding to ZnO NPs at 364 nm and 252 nm for CuO NPs.In an in vitro study, both ZnO and CuO NPs significantly (*p* < 0.05) inhibited the radial growth of *P. infestans* at all tested concentrations compared to the untreated control. The highest inhibitory effect of 100% was observed with ZnO and CuO NPs at 30 mg/L. A lower inhibition of 60.4% was observed with 10 mg/L CuO NPs. Under greenhouse conditions, 100 mg/L ZnO NPs was the most effective treatment for controlling potato late blight, with an efficacy of 71%. CuO NPs at 100 mg/L followed closely, with an efficacy of 69%. Based on these results, ZnO and CuO NPs are recommended as promising eco-friendly fungicides for the management and control of potato late blight after further research.

**Graphical abstract:**

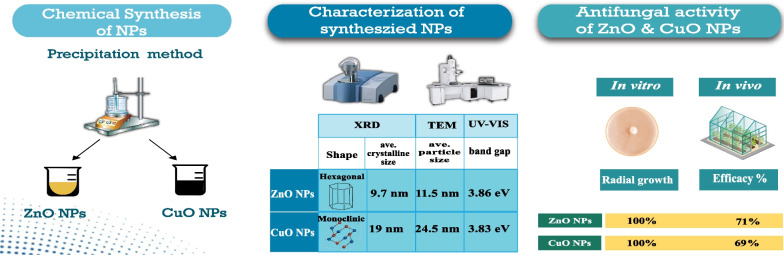

## Introduction

**Fig. 1:**
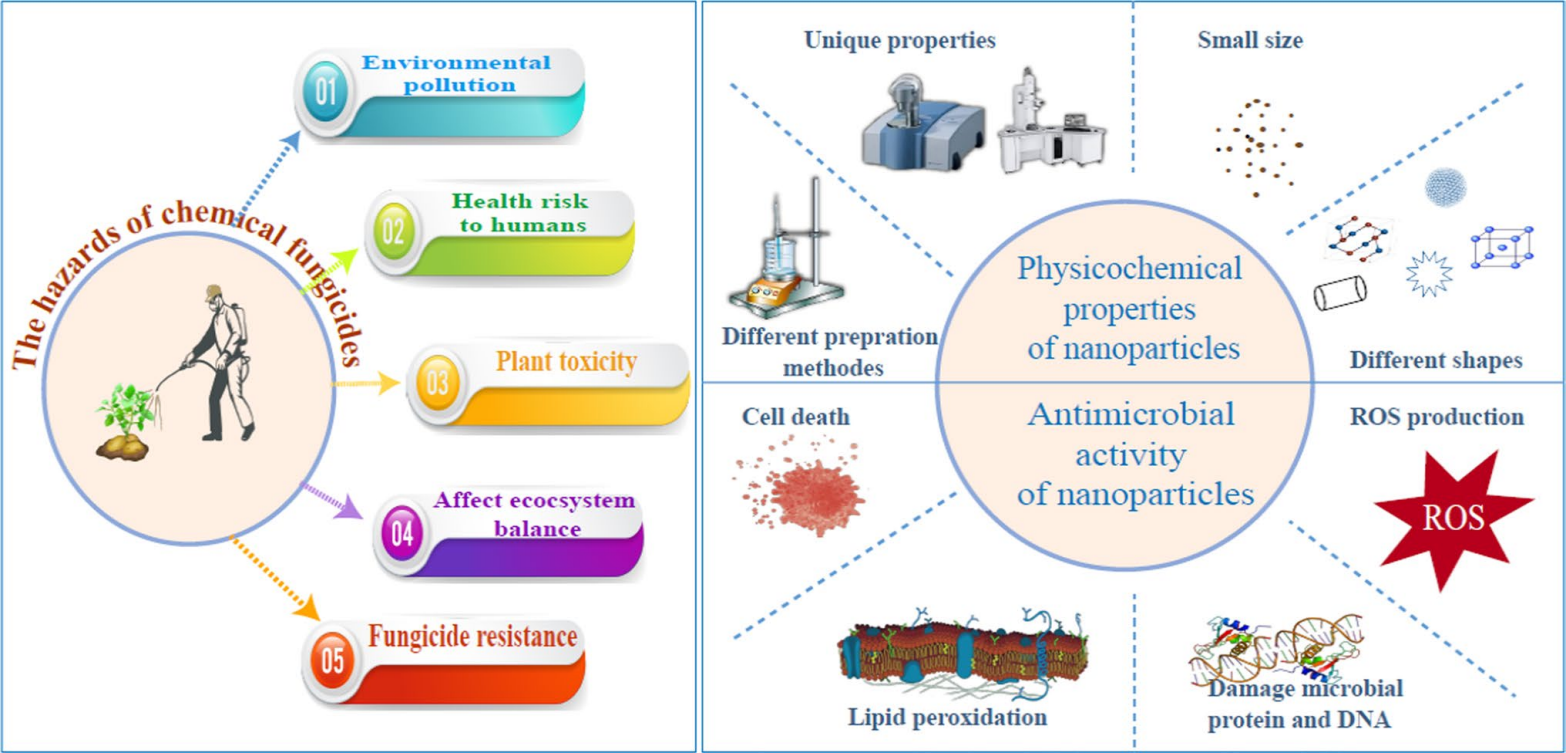
Diagram illustrating the dangers of chemical fungicides, physicochemical characteristics, and the antimicrobial effects of nanoparticles

The potato crop holds significant historical, social, and economic importance in various regions around the world [1]. It has played a crucial role in terms of food security, nutrition, and population growth. However, potato productivity can be significantly reduced by various diseases. Among these diseases, potato late blight disease, caused by *Phytophthora infestans*, which is estimated to cost up to $10 billion in crop loss and management costs [2]. Chemical fungicides are commonly used to protect crops from fungal diseases. However, their excessive use has resulted in harmful effects on humans, plants, and the environment (Fig. 1) [3]. The emergence of pathogen resistance to fungicides has become a challenging issue that threatens the efficacy of highly potent commercial fungicides [4]. Therefore, it is important to explore alternative antifungal agents with novel modes of action.

Nanotechnology has the potential to revolutionize agriculture by offering a range of benefits such as improved soil health, increased crop yields, reduced reliance on toxic chemicals, and enhanced agroecosystem sustainability [5, 6]. Nanoparticles, due to their distinctive physicochemical properties and enhanced efficacy against plant pathogens, have the potential to transform conventional fungicides [7]. They offer several advantages in terms of their antimicrobial activity, such as the ability to penetrate the cell membrane and target and destroy microbes directly because of their small size [8]. Moreover, their efficacy can be further increased by customizing their size, shape, and chemical composition for particular applications [9].

Metal oxide nanoparticles possess several advantages that render them promising materials for the development of new antimicrobial agents [10]. These benefits include a high surface-area-to-volume ratio, unique optical, electronic, and magnetic properties, high stability, simple preparation methods, and easy adjustment to the desired size, shape, and porosity [11]. They have been demonstrated to be lethal to a range of pathogenic organisms, including bacteria [12], fungi [13], oomycetes [14], and viruses [15]. Metal oxide nanoparticles exhibit promising antifungal activity by overproducing reactive oxygen species (ROS) [16], such as hydroxyl groups, superoxide anions, and hydrogen peroxide, in the cells. This process damages microbial proteins and DNA and causes microbial membrane collapse [17, 18]. Consequently, this could potentially reduce the probability of antimicrobial resistance development.

Among a wide variety of metal oxide nanoparticles such as silver (Ag), selenium (Se), nickel (Ni), gold (Au), titanium dioxide (TiO_2_), and iron oxide (Fe_3_O_4_). However, zinc oxide (ZnO NPs) and copper oxide (CuO NPs) have attracted attention in the agricultural sector because of their unique electronic, chemical, and physical properties, low cost, low toxicity, and antimicrobial properties, as well as being important micronutrients in agriculture [19]. Nanoparticles can be synthesized using different methods, such as sol–gel, hydrothermal, mechanochemical, solvothermal, and wet chemical methods [20]. The chemical precipitation method was chosen as the approach for our study because it is fast, spontaneous, and requires simple tools and inexpensive solvents [21].

There have been several studies on the use of CuO nanoparticles in phytopathogenic fungi, either in culture media (in vitro*)* such as *Alternaria citri* [22], greenhouse conditions (in vivo*)* against *P. infestans* [23], or field conditions such as *Rhizoctonia solani* [24]. The antifungal activity of ZnO against fungi has been evaluated in vitro [25], in vivo [26], and in the field [27]. However, to the best of our knowledge, the effect of ZnO NPs on the growth of *P. infestans* has not been studied.

The aim of this study was to synthesize and characterize CuO and ZnO nanoparticles and evaluate their antifungal activities against Yemeni *P. infestans* isolate (Pi Alharethi YEM 2021) [28] under laboratory and greenhouse conditions.

## Materials and methods

The preparation of CuO and ZnO nanoparticles and their characterization were performed at the Nanotechnology and Advanced Materials Central Lab of the Agriculture Research Center, Egypt.

### Reactants

All of the reagents applied in this study were of analytical grade and were used without further purification. Zinc sulfate heptahydrate (ZnSO_4_.7H_2_O), *C* copper (II) chloride dihydrate (CuCl_2_•2H_2_O), and sodium hydroxide (NaOH) were obtained from Sigma-Aldrich, Germany. Ethanol (HPLC grade, 99.99%) and deionized water were used for solution preparation and washing throughout the experiment. All beakers were washed multiple times with Aqua Regia (HCl:HNO_3_) in a 3:1 ratio. This washing process was performed to remove any remaining impact of the chemical materials.

### Synthesis of nanoparticles

#### Synthesis of ZnO NPs

The synthesis of ZnO nanoparticles was conducted using a chemical precipitation method with a modified procedure based on the work of Goyal and Verma [29]. Initially, 36 g of zinc sulfate heptahydrate were dissolved in 250 ml of deionized distilled water through stirring for a duration of 5 min. In a separate container, 10 g of sodium hydroxide pellets were dissolved in 250 ml of deionized water, also through stirring for the same duration.

The NaOH solution was then gradually added to the zinc sulfate heptahydrate solution until the pH reached approximately 12 at room temperature, resulting in translucent white solutions. The resulting precipitate was washed with deionized water and subsequently dried in a hot-air oven at 60 °C for 24 h. Finally, the dried precipitate was finely crushed into a powder and subjected to calcination in a muffle furnace at 400 °C for a duration of two hours, utilizing a Nabertherm GmbH Model 369,396 furnace from Germany.

#### Synthesis of CuO NPs

CuO nanoparticles were synthesized using a conventional chemical precipitation process [30]. Initially, 5.4 g of sodium hydroxide pellets and 22.5 g of copper (II) chloride dihydrate were separately dissolved in ethanol. The sodium hydroxide solution was continuously stirred at 100 °C, while the copper (II) chloride dihydrate solution was stirred at room temperature. Once completely dissolved, the sodium hydroxide solution was added dropwise to the copper (II) chloride dihydrate solution with constant stirring at room temperature. The color of the solution changed from green to bluish-green and eventually to black as the reaction progressed.

The following day, the resulting precipitate was filtered using Buchner filtration and subsequently washed with ethanol and deionized water until reaching a pH of 7. The precipitate was then dried overnight in an oven at approximately 50 °C. The annealed sample was ground to obtain powdered nanoparticles, which were further subjected to calcination at 500 °C for four hours. This process produced crystalline white powders containing CuO nanoparticles.

### Nanoparticles characterization

The synthesized nanoparticles were characterized using several techniques to determine their material properties. These techniques included X-ray diffraction (XRD), which was employed to analyze the crystal structure and crystallite size of the nanoparticles; UV-spectrophotometry, which was used to investigate the optical characteristics of the synthesized nanoparticles; Fourier-transform infrared spectroscopy (FTIR) analysis, which helped identify the functional groups present in the nanoparticles; and High-resolution transmission electron microscopy (HRTEM),which was utilized to examine the surface morphology and other characteristics of the synthesized nanoparticles. These techniques provided valuable insights into the primary characterization of the material properties of the synthesized nanoparticles.

#### X-ray diffraction (XRD)

X-ray diffraction (XRD) was employed to analyze the crystal structure and determine the crystallite size of the synthesized nanoparticles. The crystal structures of zinc oxide and copper oxide were studied using an X-ray diffractometer (XPert PRO; PAN Analytical, Netherlands). with a wavelength (λ) of 1.5418 Å radiation. The powdered samples were carefully positioned on a glass slide and subjected to scanning within the angular range of 4° to 80°. The resulting diffraction patterns underwent thorough analysis using X-pert HighScore Plus (version 3, Panalytical) [31] and Origin Pro (version 9.3, OriginLab) [32] software. These analyses aimed to ascertain the crystal structure and facilitate the calculation of the average crystallite size for both CuO and ZnO nanoparticles, employing the Scherrer equation.

$$\text{D}=\text{K }\frac{\uplambda }{\text{b cos }2\uptheta }$$

where D is the average crystallite size, K is a constant equal to 0.94, λ is the wavelength of the X-ray radiation (0.154 nm), β is the full-width half-maximum of the peak (in radians), and θ is Bragg’s angle (in degrees).

The percentage of crystallinity was calculated using the peak area and Origin lab software, according to the following equation:

$${\text{Crystallinity of NPs }}\left( \% \right) = \left( {{\text{Area under the peaks}}/{\text{Area under the pattern}}} \right)*{1}00.$$

#### High-resolution transmission *electron* microscopy (HR-TEM)

High-resolution transmission electron microscopy (HRTEM) was used to analyze the size, shape, and surface morphology of the synthesized nanoparticles. HRTEM imaging was performed using HR-TEM (JEOL, JEM-2100, USA) at the Nanotech Company, Egypt. In order to prepare the sample for analysis, a small quantity of the synthesized particles was dispersed in water and subjected to sonication to disperse any agglomerates. Subsequently, a few drops of the sonicated suspension were dispersed onto a carbon-coated copper grid and allowed to dry. The grids were then loaded into the HRTEM instrument, and images were acquired at various magnifications. The acquired HRTEM images were analyzed using Image J software [33] to measure the nanoparticle size and observe any structural features, such as lattice fringes or defects.

#### Ultraviolet–visible spectroscopy (UV–Vis spectroscopy)

UV–Vis spectroscopy was utilized to investigate the optical properties and absorption characteristics of the synthesized nanometal oxides. UV–Vis measurements were performed using a [VARIAN 5000 UV–VIS-NIR, Australia] equipped with a 200–800 nm range.

Prior to analysis, a dilute solution of the synthesized nanometal oxides was prepared by dispersing a small amount of the particles in a transparent medium. The resulting aqueous solution was then placed in a quartz cuvette for measurement. Deionized water from Milli-Q served as the blank solution for baseline correction. UV–Vis spectra were obtained by measuring the absorbance of the nanoparticles at different wavelengths.

The obtained spectra were subjected to analysis using origin software to identify the absorption peaks. Additionally, the bandgap energy was calculated using Tauc’s relation [34]

$$\alpha {\text{h}}\nu = \left( {{\text{h}}\nu \, - {\text{ Eg}}} \right)^{{\text{n}}}$$

where α is the optical absorption coefficient, h is the photon energy, A is a constant, and n is the exponent that determines the type of electronic transition that causes the absorption and can take the values 2 or 1/2, depending on whether the transition is direct or indirect.

#### Fourier-transform infrared spectroscopy (FT-IR)

Fourier-transform infrared spectroscopy (FT-IR) is an analytical technique that can be used to identify functional groups present in materials. The technique involves measuring the absorption of infrared radiation by the sample material, which provides valuable information about its chemical composition and structure. FTIR measurements were performed using a Bruker FTIR spectrophotometer from Germany, with infrared scanning performed in the range of 4000–400 cm^−1^. The sample preparation involved grinding the nanoparticle powder into a fine consistency and pressing it into pellet form. The pellets were then mounted on an IR transparent window for spectral measurement.

### Fungal strain and culture conditions

The blighted samples were collected from the areas of the Seed Potato Production Center (SPPC) in Ibb Governorate in Yemen. The *P. infestans* strain Pi Alharethi YEM 2021, identified by its GenBank accession number OQ119020, was utilized in this study. It underwent morphological, microscopic, and genetic identification, and was tested on both V8 Agar and Rye Agar [28, 35]. V8 Agar, which exhibited favorable growth conditions, was selected for culturing *P. infestans*. The isolate was then transferred onto V8 agar supplemented with 100 mg/L ampicillin, 20 mg/L rifampicin, and 50 mg/L nystatin [36].

### In vitro assay of the antifungal activity of synthetic NPs

The method employed by Joshi et al*.* [37] was utilized to evaluate the antifungal properties of ZnO and CuO nanoparticles (NPs). The concentrations selected for assessing the antifungal efficacy of ZnO and CuO nanoparticles in vitro were 10, 20, and 30 mg/L. These concentrations were established following a review of previous literature and preliminary experiments on the antifungal activity of ZnO and CuO nanoparticles [38, 39]. The antifungal activity of ZnO and CuO NPs was determined by measuring the extent of growth suppression on *P. infestans* in comparison to an untreated control. In this experiment, V8 medium was melted and combined with metal NP solutions at varying concentrations (10, 20, and 30 mg/L) in Petri dishes, resulting in a final volume of 15 mL. Subsequently, a 0.5 cm diameter of a 10-day-old pure culture of *P. infestans* isolates (Pi Alharethi YEM2021) was placed individually at the center of each Petri dishe. The dishes were covered with parafilm and incubated at a temperature of 18 °C [40]. Each NPs concentration was replicated three times to ensure statistical robustness. The determination of the replication was aided by the GPower program, which considered a 95% confidence level when comparing the means of a previous study. After a period of two weeks, the growth diameters and inhibitory effects of the control and metal oxide NPs were measured. The percentage of growth inhibition was calculated using the following formula:

$$\text{Growth}\, \text{inhibition }\left(\text{\%}\right)=\frac{\text{D}1-\text{D}2}{\text{D}1} \times 100$$

where D1 and D2 are the colony diameters of the control and samples containing metal oxide NPs, respectively.

### Greenhouse experimental design

The efficacy of ZnO and CuO NPs was studied in pots under greenhouse “in vivo” conditions at Biological Science Department in Sana’a University between March and June 2022. The experiment was designed using a completely randomized design with three replicates. Potato seed tubers were planted in plastic pots (50 cm diameter, 25 cm height) filled with sandy loam soil, with one tuber per pot.

The greenhouse conditions maintained a temperature range of 18–22 °C and a relative humidity of 80–85%. The experiment comprised three groups: a control group that was sprayed with an inoculum of *P. infestans* without exposure to nanoparticles, a ZnO NPs group where three different concentrations of ZnO NPs (25, 50, and 100 mg/L) were applied, with each concentration having three replicates, and a CuO NPs group where three different concentrations of CuO NPs (25, 50, and 100 mg/L) were applied, again with each concentration having three replicates.

On day 34 of planting, before inoculation, whole plants were sprayed with ZnO and CuO NPs at concentrations of 25, 50, and 100 mg/L using a hand sprayer (30 mL per plant). On day 37 after planting, whole plants were sprayed with an inoculum of *P. infestans* (Pi Alharethi YEM 2021) at a concentration of 5 × 104 sporangia per ml. On day 40 after inoculation, whole plants were sprayed with ZnO and CuO NPs until the leaves were completely wet, allowing the solution to run off the leaves.

All pots were irrigated three times throughout the seven days, and transparent plastic bags were placed over the inoculated plants for 48 h to maintain high relative humidity and promote fungal infection [41]. Disease severity was determined at 7, 14, and 21 days post-inoculation based on the Henfling modified disease estimation scale for late blight of potatoes [42] (Table 1).

**Table 1:** Henfling modified disease estimation scale for late blight of potatoes

Grade	% Incidence	Nature of Infection
0	0	No disease
1	10%	Small lesions on the inoculated point, with the lesion area less than 10% of the whole leaflet
3	10 and 20%	Lesions area between 10 and 20% of the whole leaflet
5	20 and 30%	Lesion area between 20 and 30% of the whole leaflet
7	30 and 60%	Lesion area between 30 and 60%
9	Over 60%	Lesion area over 60% of the whole leaflet

Disease severity has been used to determine a disease severity index (DSI) on a percentage basis, were

$$DSI(\%)=\frac{Sum\, of\, individual\, ratings }{No.\,of\, plants\,examindX \,Max.disease\, scale }\times 100$$

The area under the disease progression curve (AUDPC) was calculated according to Madden et al*.* [43] as follows:

$$\text{AUDPC}={\sum }_{i=1}^{n-1}\frac{\left({y}_{i }+{y}_{i+1}\right)}{2}\left({t}_{i+1 }-{t}_{i}\right)$$

where “t” is the time in days at the ith observation, “y” is an assessment of a disease at the i th observation, and “n” is the total number of observations.

The efficacy of ZnO and CuO NPs was calculated using Rewal and Jhooty's [44] formula.

$$Efficacy=\frac{Control -T }{C }X 100$$

C is the percentage of infection in the control group, and T is the percentage of infection in the treatment group.

### Statistical analysis

All measured data were statistically analyzed using the Statistical Package for Social Sciences (SPSS) version 25. A one-way ANOVA was computed using the least significant difference (LSD) at the 5% probability level to compare mean values, followed by Tukey’s post-hoc test.

## Results

### XRD analysis

**Fig. 2:**
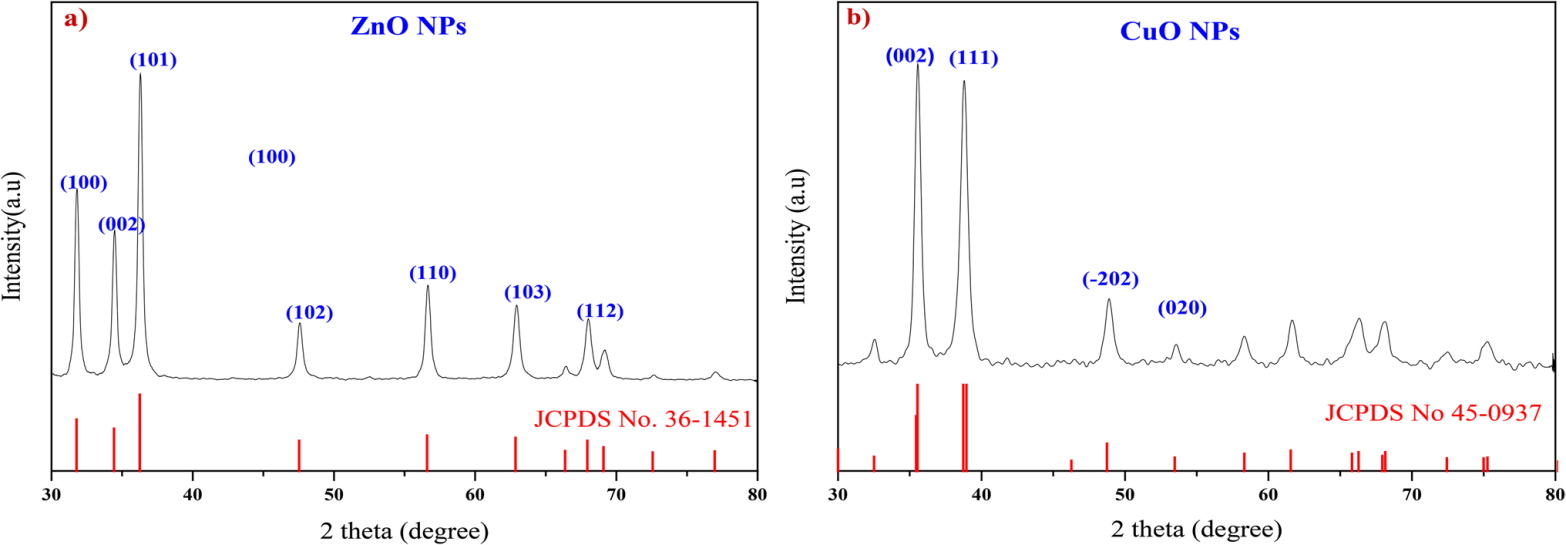
XRD Diffraction Pattern for **a**: ZnO NPs and **b** CuO NPs

X-ray diffraction (XRD) analysis was performed to investigate the structural properties of the ZnO and CuO nanoparticles synthesized using chemical co-precipitation. The XRD pattern for ZnO (Fig. 2a) reveals distinct peaks at the following angles: 31.8°, 34.46°, 36.29°, 47.65°, 56.6°, and 62.9°. These peaks correspond to the lattice planes (100), (002), (101), (102), (110), (103), and (112), respectively. The ZnO (101) diffraction peak is much stronger than other peaks. This indicates that the formed ZnO nanocrystals have a preferential crystallographic (101) orientation. These findings confirm that the synthesized ZnO nanoparticles possess a wurtzite crystalline structure with lattice constants of a = b = 3.2499 Å and c = 5.2066 Å. These patterns align with those in the Joint Committee on Powder Diffraction Standards (JCPDS) card No. 36–1451.Conversely, the XRD pattern for CuO (Fig. 2b) exhibits sharp peaks at 35.6° and 38.7°, corresponding to the lattice planes (002) and (111), respectively. The CuO (002) and (111) diffraction peaks are much stronger than other peaks. This indicates that the formed CuO nanocrystals have a preferential crystallographic (002) and (111) orientation. This indicates that the synthesized CuO nanoparticles possess a monoclinic crystalline structure with lattice constants of a = 4.68 Å, b = 3.42 Å, and c = 5.13 Å, as per JCPDS card No. 45–0937. The presence of high-intensity peaks in both ZnO and CuO patterns suggests that both materials were formed in highly crystalline structures. Furthermore, no additional peaks were detected in the XRD analysis, confirming the formation of pure ZnO and CuO phases. Using Debye Scherrer's equation, the average crystalline sizes were determined to be 9.7 nm for ZnO nanoparticles and 19 nm for CuO nanoparticles. The observed crystallinity of ZnO NPs was 99%, while that of CuO NPs was 91%, indicating that both materials exhibited highly crystalline structures.

### High-resolution transmission *electron* microscopy (HR-TEM)

**Fig. 3:**
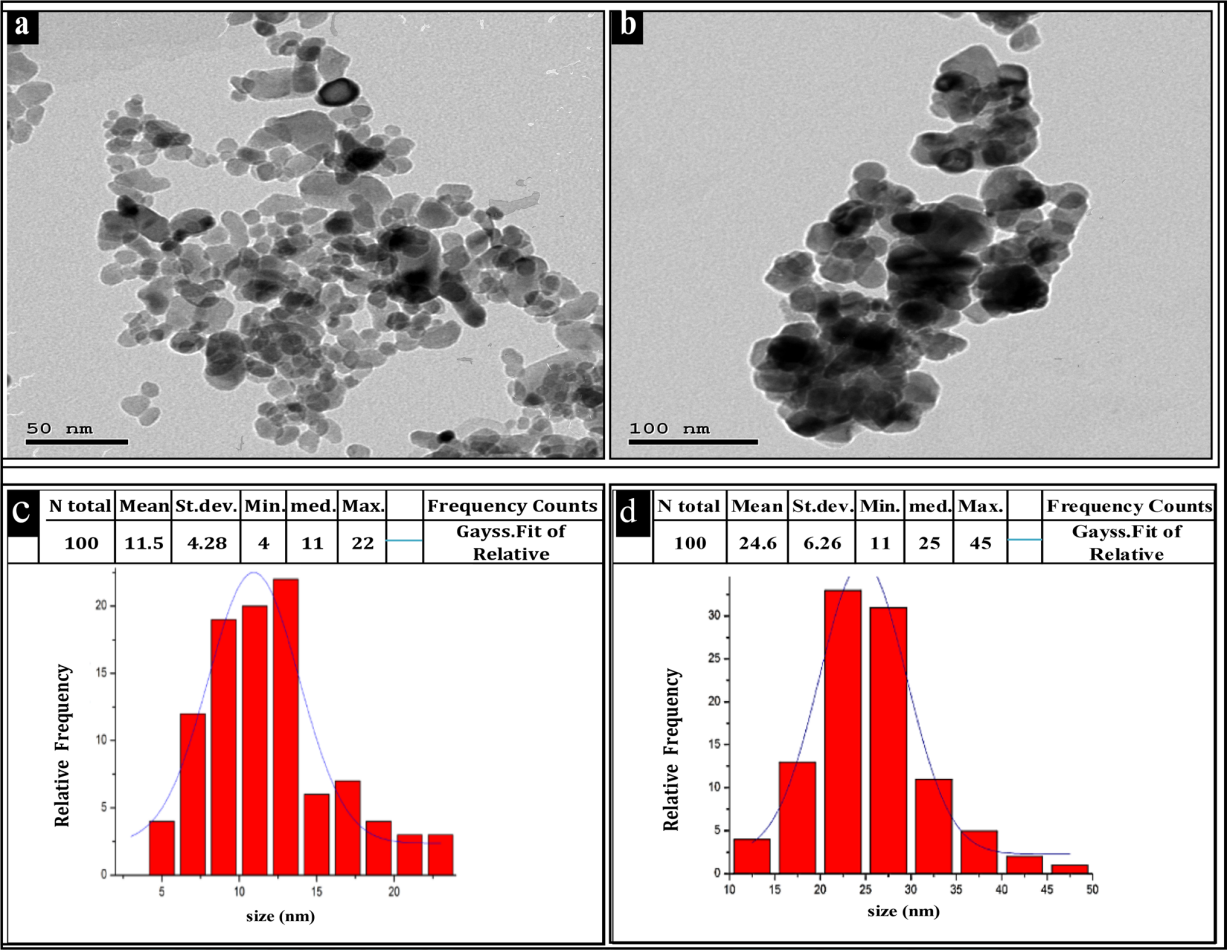
TEM analysis: **a** ZnO NPs TEM image; **b** CuO NPs TEM image; **c** ZnO NPs particle size distribution histogram; **d** CuO NPs particle size distribution histogram

The HR-TEM analysis provided insights into the size, shape, and crystallinity of the synthesized ZnO and CuO nanoparticles. The TEM images in (Fig. 3a and b) revealed that these nanoparticles exhibit a spherical shape and fall within a size range of 4–25 nm for ZnO and 11–25 nm for CuO. Histograms in (Fig. 3c and d) depict the particle size distributions for ZnO NPs and CuO NPs, respectively. The mean sizes of the synthesized NPs were 11.5 nm for ZnO and 24.5 nm for CuO NPs. Notably, ZnO NPs with sizes of 9 nm, 11 nm, and 12.5 nm constituted 18%, 19%, and 22% of the total, respectively. Conversely, the largest measured ZnO NPs were approximately 25.5 nm in diameter, accounting for only 4% of the total. For CuO NPs, the majority fell within the sizes of 17.5 nm, 22.5 nm, and 27.5 nm, representing 13%, 35%, and 30% of the total, respectively. The largest CuO NPs measured approximately 42.5 nm, constituting only 2% of the total NPs.

**Fig. 4:**
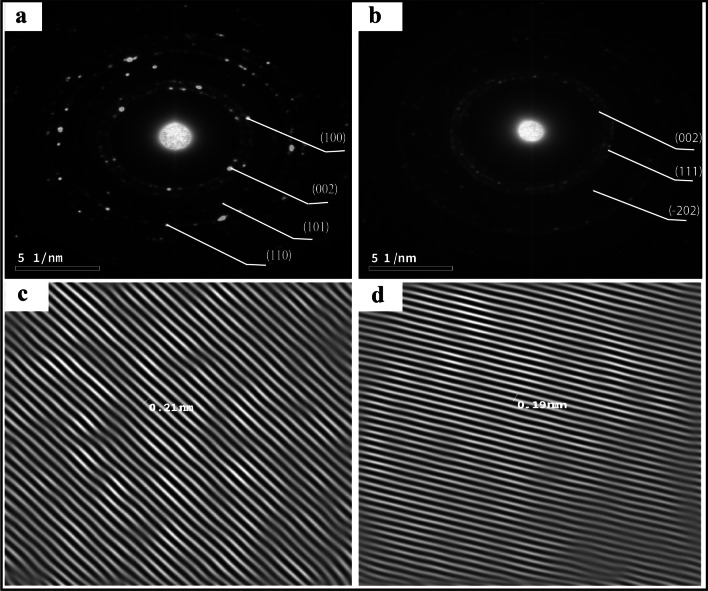
HRTEM image of **a** ZnO NPs; **b** CuO NPs; **c** The inter-planar distance of ZnO NPs; **d** The inter-planar distance of CuO NPs

Figure 4a and b showcase extremely high-resolution TEM (HRTEM) images of pure ZnO and CuO NPs, respectively. The inter-planar distances (d-spacing) were approximately 0.21 for ZnO NPs (Fig. 4c) and 0.19 for CuO NPs (Fig. 4d). The relative intensity of SAED rings corresponds to the XRD pattern peaks, indicating that ZnO NPs possess a hexagonal wurtzite.

### UV–Vis spectroscopy analysis

**Fig. 5:**
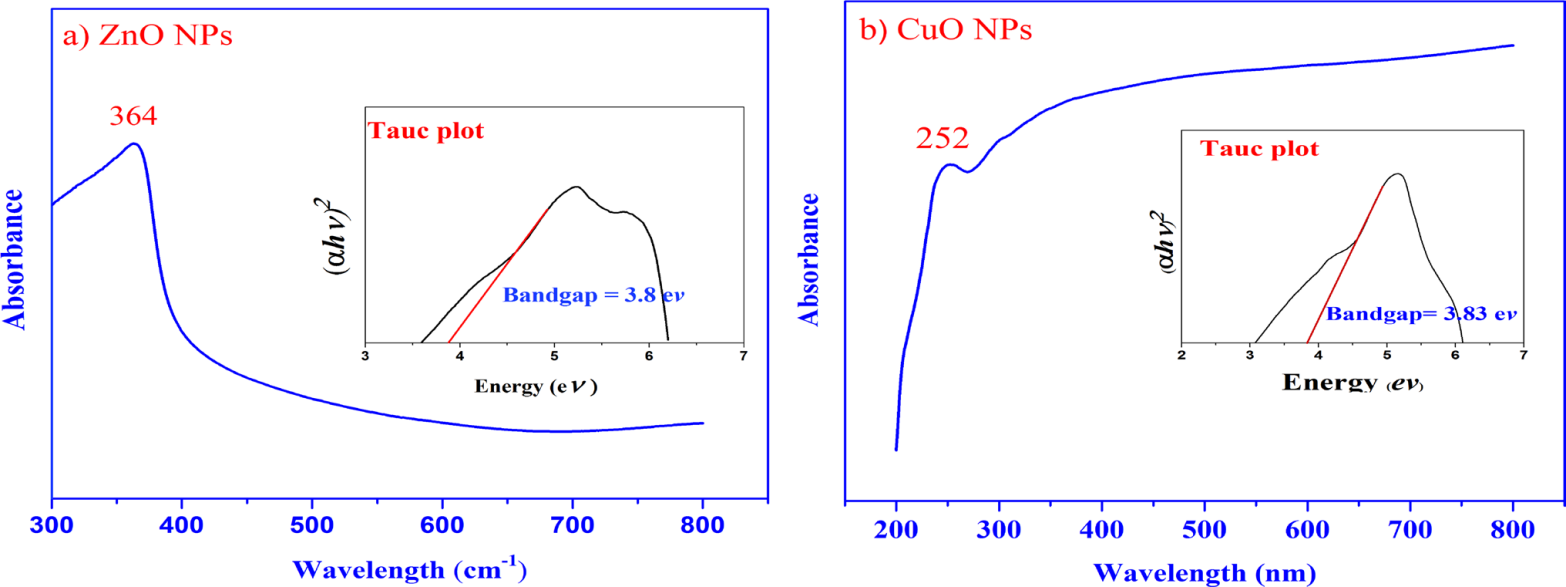
UV–Vis spectra and Tauc plots of: **a** ZnO and **b** CuO nanoparticles. Inset in **a** is the Tauc plots of ZnO NPs.Inset in **b** is the Tauc plots of CuO NPs

UV–Vis analysis was performed on the synthesized ZnO and CuO nanoparticles to investigate their optical properties. The analysis revealed characteristic absorption peaks at 364 nm for ZnO NPs (Fig. 5a) and 252 nm for CuO NPs (Fig. 5b). To determine the optical band gap (Eg) of the nanoparticles, Tauc's relation [34] was employed:

$$\alpha {\text{h}}\nu = \left( {{\text{h}}\nu \, - {\text{ Eg}}} \right)^{{\text{n}}}$$

where α is the optical absorption coefficient, h is the photon energy, A is a constant, and n is the exponent that determines the type of electronic transition that causes the absorption and can take the values 2 or 1/2, depending on whether the transition is direct or indirect.

Tauc's relation is a widely used method to determine the band gap energy from the absorption spectra. By plotting the absorption coefficient (α) multiplied by the photon energy (hν) as a function of the photon energy, the band gap energy can be estimated from the intercept on the energy axis, with the transition data offering the most accurate linear fit in the band edge region for n = 2. Using this relation, the optical band gap of the synthesized ZnO nanoparticles was calculated to be 3.86 eV (inset of Fig. 5a), while for CuO nanoparticles, it was determined to be 3.83 eV (inset of Fig. 5b).

### The Fourier-transform infrared spectroscopy (FT-IR)

FT-IR analysis was conducted to investigate the molecular vibrations and functional groups present in the synthesized ZnO and CuO nanoparticles.

**Fig. 6:**
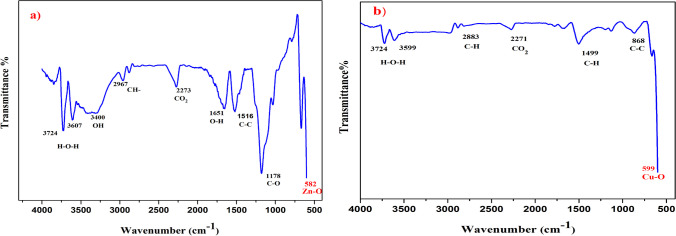
The FTIR spectra of: **a** ZnO and **b** CuO nanoparticles

In the FTIR spectrum of ZnO nanoparticles (Fig. 6a), several significant absorption peaks were observed. These include peaks at 3724 cm^−1^, 3607 cm^−1^, 3400 cm^−1^, 2967 cm^−1^, 2875 cm^−1^, 2273 cm^−1^, 1651 cm^−1^, 1516 cm^−1^, 1178 cm^−1^, and 582 cm^−1^.

The absorption peaks at 3724 cm^−1^ and 3607 cm^−1^ were attributed to the O–H bending vibration, indicating the presence of a small amount of H_2_O in the ZnO NPs. The broad band at 3400 cm^−1^ and the peak at 1651 cm^−1^ were associated with the OH-stretching vibration. The absorption peaks at 2967 cm^−1^ and 2875 cm^−1^ were assigned to C-H stretching vibrations. The absorption peak at 2273 cm^−1^ indicated the presence of CO_2_ molecules in the surrounding air. The peaks at 1516 cm^−1^ and 1178 cm^−1^ corresponded to the stretching vibrations of the C–C–C and C–O bonds, respectively. Finally, the peak at 582 cm^−1^ in the fingerprint region confirmed the Zn–O stretching vibration, providing evidence for the formation of the wurtzite structure of the ZnO NPs.

In the FTIR spectrum of CuO nanoparticles (Fig. 6b), the absorption band characteristics were observed. The peak at 599 cm^−1^ indicated the formation of a CuO nanostructure. The peaks around 3724 cm^−1^ and 3599 cm^−1^ were attributed to the O–H bending vibration caused by atmospheric moisture. The peak at 2271 cm^−1^ represented the presence of CO_2_ molecules from the surrounding air. The vibration bands at 2883 cm^−1^ and 1499 cm^−1^ were assigned to C-H stretching.

### In vitro Antifungal activity of ZnO and CuO NPs against *P. infestans*

**Fig. 7:**
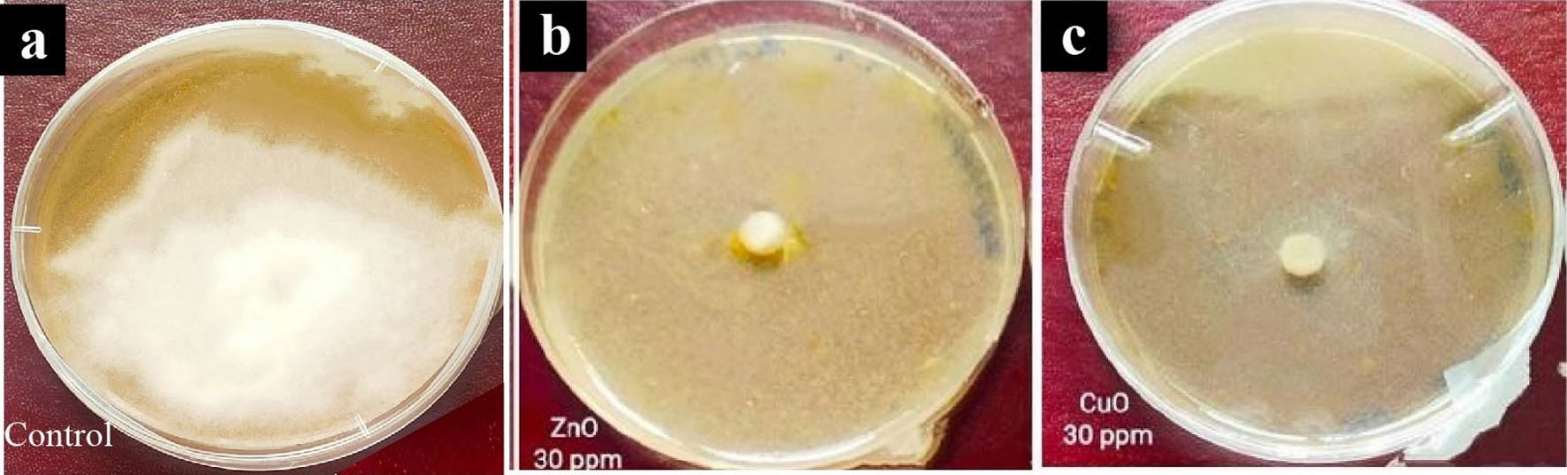
Efficacy of CuO and ZnO NPs at concentration of 30 mg/L, on the mycelium growth of *P. infestans* isolate compared to untreated control. **a** Control, **b** ZnO NPs, and **c** CuO NPs

The antifungal activity of ZnO and CuO nanoparticles (NPs) against the *P. infestans* isolate (Pi Alharethi YEM2021) cultivated on V8 medium was evaluated using different concentrations of ZnO and CuO NPs (10, 20, and 30 mg/L) compared to the control. After two weeks of incubation, the growth inhibition of *P. infestans* was assessed, and the results are summarized in Table 2 and Fig. 7.

**Table 2:** In vitro radial growth and inhibition of *P. infestans* by CuO and ZnO nanoparticles

Treatment	Concentration (mg/l)	Radial growth (mm)*	Growth Inhibition %
ZnO	10	1.67 ± 0.17^**bc**^	64.2
	20	0.97 ± 0.14^**d**^	79.1
	30	0.0^**e**^	100
CuO	10	1.85 ± 0.1^**b**^	60.4
	20	1.11 ± 0.14 cd	76.3
	30	0.0 ^**e**^	100
Control		4.67 ± 0.18^a^	0.00

*The radial growth was performed on the isolate Pi Alharethi YEM 2021 that obtained from the microbial culture collection at Ibb,Yemen

Mean of the radial growth followed by ± standard error

Statistical comparisons were made within a single column using Tukey LSD test at P ≤ 0.05

Means followed by different letters are significantly different (P = 0.05), whereas means followed by the same letter are not significantly different

The highest concentration (30 mg/L) of ZnO and CuO NPs achieved a remarkable 100% inhibition of the radial growth of *P. infestans*. At 20 mg/L, ZnO and CuO NPs exhibited substantial growth inhibition percentages of 79.1% and 76.3%, respectively. Similarly, at 10 mg/L, ZnO and CuO NPs demonstrated notable inhibition percentages of 64.2% and 60.4%, respectively. These findings indicate that the antifungal activity of ZnO and CuO NPs is concentration-dependent, with a positive correlation between efficacy and concentration.

Statistical analysis revealed a significant difference between all concentrations of ZnO and CuO NPs when compared to the control group. Moreover, a statistically significant difference (P < 0.05) was observed between the concentrations of 10 mg/L ZnO NPs and 20 mg/L ZnO NPs, as well as between 20 mg/L ZnO NPs and 30 mg/L ZnO NPs. However, among all the different concentrations of CuO NPs, there was no statistically significant difference observed between the similar concentrations of ZnO and CuO NPs.

### Efficacy of ZnO and CuO NPs against *P. infestans* under greenhouse conditions

The efficacy of ZnO and CuO nanoparticles (NPs) in controlling late blight disease in greenhouse potato plants was evaluated, and the results are presented in Table 3. The treatment with ZnO NPs at a concentration of 100 mg/L demonstrated the highest inhibitory effect against *P. infestans*, with an efficacy of 70.64%. CuO NPs at 100 mg/L showed a slightly lower efficacy of 68.8%. Similarly, ZnO NPs at 50 mg/L and CuO NPs at 50 mg/L exhibited efficacy percentages of 47% and 46%, respectively, when compared to the untreated control group.

**Table 3:** The effect of ZnO and CuO NPs on *P. infestans* isolate (Pi Alharethi YEM) on potato plants in terms of disease severity%, efficacy%, and AUDPC under greenhouse conditions

Treatment	Concentration (mg/l)	Disease severity%				Efficacy%	AUDPC
		0^Φ^	7 days	14 days	21 days*		
ZnO	25	3.5 ± 0.14	18.7 ± 0.37	29.7 ± 0.4	50.5 ± 0.59 ^a^	7.34 ± 1.13	527.8 ± 6.86^b^
	50	3.2 ± 0.15	15.7 ± 0.4	23.4 ± 0.56	28.9 ± 0.93 ^b^	46.97 ± 1.31	386.8 ± 9.48^c^
	100	3.4 ± 0.11	10.9 ± 0.32	14 ± 0.34	16 ± 0.69 ^c^	70.64 ± 1.18	243.1 ± 6.73^d^
CuO	25	3.6 ± 0.13	19.2 ± 0.26	29.3 ± 0.42	50.7 ± 0.98 ^a^	6.97 ± 1.34	530 ± 8.47^b^
	50	3.4 ± 0.12	15.4 ± 0.34	24.1 ± 0.43	29.4 ± 1.08 ^b^	46.05 ± 1.4	391.6 ± 9.34^c^
	100	3.3 ± 0.13	11.2 ± 0.34	14.2 ± 0.47	17 ± 0.86 ^c^	68.8 ± 1.27	249.2 ± 8.37^d^
Control		3.4 ± 0.16	20.3 ± 0.4	31.4 ± 0.52	54.5 ± 1.0 ^a^	0	565.2 ± 9.61^a^

^Φ^First day for the application of the tested treatments under greenhouse conditions followed by 7 days intervals

*Means followed by different letters are significantly different (*P* = 0.05), whereas means followed by the same letter are not significantly different. Mean visual disease severity rating followed by ± standard error. Statistical comparisons were made within a single column using Tukey LSD test at *P* ≤ 0.05

AUDPC = area under the disease progress curve

However, lower concentrations (25 mg/L) of both ZnO and CuO NPs did not significantly inhibit the severity of late blight compared to the control group. It should be noted that the inhibitory effect of both ZnO and CuO NPs increased gradually with increasing concentrations, indicating a concentration-dependent response.

Statistical analysis indicated no significant difference in the prevention of *P. infestans* growth between similar concentrations of ZnO and CuO NPs in the two groups. This suggests that both types of nanoparticles exhibited comparable efficacy in controlling late blight disease.

Furthermore, the control treatment had the highest Area Under the Disease Progress Curve (AUDPC) value of 565.2, indicating the highest disease severity. In contrast, the lowest AUDPC value of 243.1 was observed in the treatment with ZnO NPs at 100 mg/L. This was followed by CuO NPs at 100 mg/L, ZnO NPs at 50 mg/L, and CuO NPs at 50 mg/L, with AUDPC values of 249.2, 386.8, and 391.6, respectively.

## Discussion

As a result of the increasing challenge posed by fungal resistance to traditional fungicides, there is a critical drive to create innovative, eco-friendly methods for controlling fungal diseases in plants. Metal oxide nanoparticles are considered a promising alternative for controlling phytopathogenic fungi in agriculture [45, 46].

The findings of our study underscore the promising antifungal properties of synthesized zinc oxide (ZnO) and copper oxide (CuO) nanoparticles (NPs) in the context of late blight disease management in potatoes. Both in vitro and in vivo investigations revealed encouraging results, suggesting that these NPs could serve as effective agents against *Phytophthora infestans,* the causal agent of late blight.

Our observations align with previous research by Giannousi et al*.* [23]*,* who demonstrated the efficacy of Cu-based NPs in mitigating late blight disease in tomatoes. Furthermore, the utilization of ZnO-NPs as novel antifungal agents has been extensively explored in the literature [47–49]. Surprisingly, despite the growing interest in ZnO NPs, there are no documented reports specifically investigating their effectiveness against potato late blight caused by *P. infestans.* Our study bridges this gap by providing evidence of their potential utility in disease management.

Metal oxide nanoparticles have garnered attention for their antifungal activity, including *P. infestans.* Elsharkawy et al*.* [39] demonstrated the inhibitory effect of Ag_2_O and Ag_2_O /TiO_2_ against *P. infestans* across various conditions, including laboratory, greenhouse, and field settings. Similarly, Khan et al*.* [38] highlighted the efficiency of iron oxide NPs in controlling the pathogen in vitro. Additionally, Wang et al*.* [50] reported that magnesium oxide nanoparticles (MgO NPs) effectively protect potatoes from late blight pathogens even at a low dosage of 50 µg/mL.

The X-ray diffraction (XRD) analysis successfully confirmed the crystalline nature of both ZnO and CuO nanoparticles. This conclusion arises from the presence of intense peaks in the respective XRD patterns for each material. Importantly, the average crystalline sizes determined in this study were 9.7 nm for ZnO nanoparticles and 19 nm for CuO nanoparticles. These values align with previous research, consistent with the findings of Bhandari et al*.* [51] for ZnO NPs and Amin et al*.* [52] for CuO NPs.

TEM analysis revealed the formation of stable, predominantly quasi-spherical ZnO and CuO NPs, with some degree of agglomeration. The average particle sizes were determined to be 11.5 nm for ZnO NPs and 24.5 nm for CuO NPs. These findings are consistent with previous studies conducted on ZnO NPs by Raliya and Tarafdar [53] and Shamhari et al*.* [54], as well as on CuO NPs by Dagher et al*.* [55] and Wongrakpanich et al. [56].

The diffraction rings observed in the SAED image correspond to the peaks observed in the XRD pattern, providing evidence for the hexagonal wurtzite structure of ZnO nanoparticles and the monoclinic crystalline structure of CuO NPs. The shape and size of nanoparticles are very important for their activity against microbial pathogens. NPs with smaller sizes can easily enter the cell wall of microbes and disrupt the cell walls, followed by blocking replication[57]

The Fourier-transform infrared spectroscopy (FTIR) analysis conducted on the synthesized ZnO NPs indicated an absorption peak at a wavenumber of 582 cm^−1^. Similarly, the FTIR analysis of CuO NPs revealed an absorption peak at a wavenumber of 599 cm^−1^. These findings are in agreement with previous studies that have investigated ZnO and CuO NPs, which have likewise documented absorption peaks falling within this specific range [58, 59].

Additionally, FTIR analysis indicated the presence of H_2_O and CO_2_ impurities originating from the surrounding environment. This occurrence is attributed to the oxidation of the nanoparticle surface, leading to the formation of metal hydroxides or oxide-hydroxide species capable of absorbing water molecules.

The detection of CO_2_ impurities, particularly near 2273 cm-1 in both ZnO and CuO nanoparticles, suggests exposure to air or moisture, consistent with findings from studies by Hlaing et al. [60] and Varghese et al. [61]. Additionally, peaks observed around 3724 cm-1 in both ZnO and CuO spectra were attributed to O–H bending vibrations induced by atmospheric moisture, as supported by earlier research [62, 63].

The ultraviolet–visible (UV–Vis) spectra analysis exhibited absorption peaks at 364 nm, corresponding to the ZnO NPs, and at 252 nm, corresponding to the CuO NPs. These findings align with prior investigations that have reported comparable absorption peaks within this wavelength range.

The band gap value of nanoparticles refers to the energy difference between the valence band and the conduction band of the material. The sharp absorption peaks observed at 364 nm and 252 nm confirm the formation of ZnO and CuO nanoparticles (NPs). Additionally, there was a significant blueshift in the excitonic absorption of the prepared NPs compared to that of the bulk ones. This blueshift in the excitation absorption clearly indicates the quantum confinement property of NPs [64, 65].

Notably, the calculated band gaps for the ZnO NPs and CuO NPs were determined to be 3.86 eV and 3.83 eV, respectively. These values are larger than the reported band gap values for bulk ZnO (3.37 eV) and CuO (3.5 eV) [66, 67]. The increase in band gap can be attributed to the reduction in particle size of the ZnO NPs and CuO NPs [68]. This phenomenon is caused by electron transitions from the valence band to the conduction band [69]. A higher band gap value is advantageous for photocatalytic applications. When exposed to UV light, both ZnO and CuO NPs demonstrate increased photocatalytic activity, resulting in the generation of reactive oxygen species (ROS) [70].

**Fig. 8:**
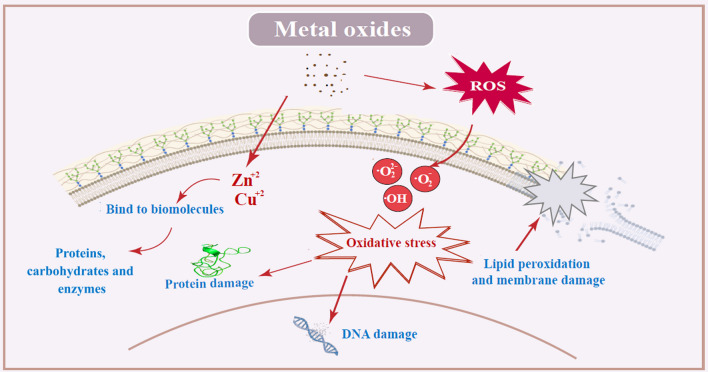
Schematic diagram of possible mechanism of action of Metal oxides nanoparticles against *P. infestans*

Figure 8 provides a comprehensive overview of the mechanisms of action associated with metal oxide nanoparticles. These mechanisms can be summarized as follows: First, metal oxide nanoparticles interfere with the integrity of cell membranes, potentially compromising cellular functions [71]. Second, these nanoparticles generate reactive oxygen species (ROS) like superoxide, hydroxyl ions, hydroxyl radicals and hydrogen peroxide, which contribute to oxidative stress and cellular damage [72, 73]. Third, metal oxide nanoparticles have the ability to bind to proteins and DNA, causing structural alterations and functional impairments [18]. Lastly, metal oxide nanoparticles, such as ZnO NPs and CuO NPs, release metal ions (Zn^2+^ and Cu^2+^), which can further influence cellular processes [74].

In our in vitro study, both CuO NPs and ZnO NPs exhibited significant antifungal efficacy across all tested concentrations, effectively inhibiting the average radial growth of *P. infestans* compared to the control group. Notably, the highest antifungal activity was observed at a concentration of 30 mg/L for both types of nanoparticles, with the effectiveness diminishing as the concentrations decreased.

The assessment of nanomaterials in greenhouse experiments holds paramount significance. These evaluations offer valuable insights into the potential real-world applications and environmental consequences of nanomaterials. Greenhouse experiments serve as essential platforms for investigating the effects of nanomaterials on plants under precisely controlled conditions. By manipulating variables such as temperature, humidity, and light, researchers can thoroughly assess the efficacy of nanomaterials in safeguarding plants against pests, diseases, and environmental stressors.

In our greenhouse experiments, we conducted applications of ZnO and CuO nanoparticles (NPs) both before and after inoculation with the pathogen. The results demonstrated a significant reduction in disease severity when ZnO NPs were applied at a concentration of 100 mg/L, exhibiting an efficacy percentage of 70.64% against *P. infestans* compared to the control group. Similarly, CuO NPs at 100 mg/L also exhibited a significant reduction in disease severity, with an efficacy of 69%. This aligns with previous research by Elmer et al*.* [75], which reported the successful reduction of various fungal diseases, including tomato Fusarium wilt and Verticillium wilt, through the use of ZnO NPs and CuO NPs.

Additionally, Ritmontree and Kongtragoul [76] found ZnO NPs to be effective against *Phytophthora palmivora*. Furthermore, in greenhouse trials, Chen et al*.* [77] demonstrated that the application of CuO NPs at 100 mg/L effectively reduced *Phytophthora nicotianae.*

**Fig. 9:**
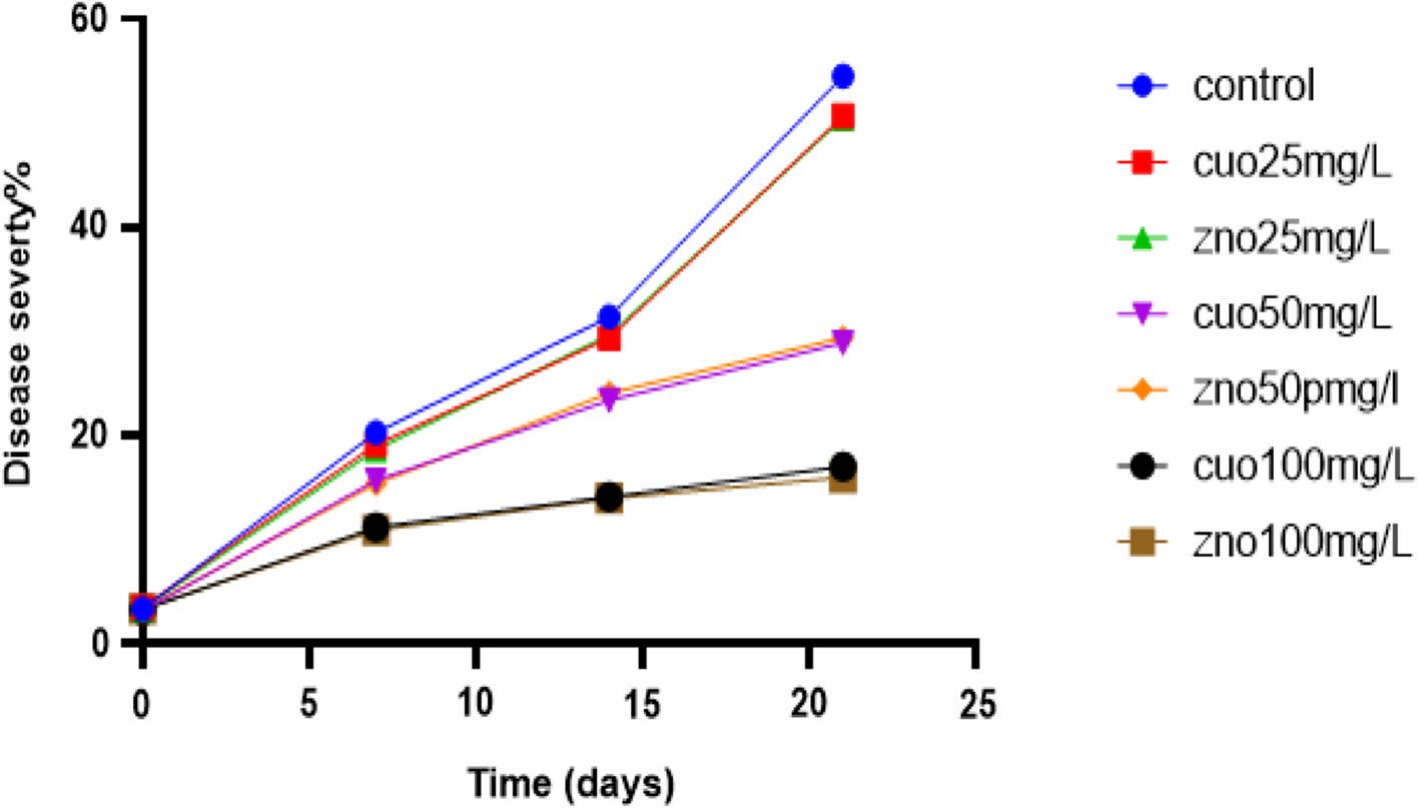
Effect of treatments on late blight severity rate over time in greenhouse conditions

Moreover, as indicated in Fig. 9, the application of ZnO NPs at 100 mg/L displayed the lowest increase rate in disease severity percentage over time, followed by CuO NPs at 100 mg/L, ZnO NPs at 50 mg/L, CuO NPs at 50 mg/L, ZnO NPs at 25 mg/L, and CuO NPs at 25 mg/L, respectively.

The results indicate that the percentage of inhibition of *P. infestans* growth increased with higher concentrations of ZnO and CuO nanoparticles (NPs). Additionally, it was observed that ZnO NPs with a smaller particle size (11.5 nm) exhibited the highest inhibition compared to CuO NPs (24.5 nm). This finding is consistent with studies by Sirelkhatim et al*.* [71] and Lai et al. [78], which suggest that the size of nanomaterials plays a crucial role in their effect on microbes. Smaller NPs have been found to possess greater antifungal activity than larger NPs.

This can be attributed to their larger surface-area-to-volume ratio, which enhances binding at various target sites on the microbial cell membrane, leading to disruption of cell integrity, the release of metal ions (Zn^2+^ and Cu^2+^) [74], and the generation of reactive oxygen species (ROS) [16]. The ROS produced may include hydroxyl radicals (OH•), superoxide ions (O2•), and singlet oxygen (1O2), which induce lipid peroxidation and ultimately result in cell death [17]. Tryfon et al. [79] demonstrated that the synthesized ZnO@OAm NRs resulted in a decrease in the efficiency of the oxygen-evolving complex (OEC), accompanied by an increase in reactive oxygen species (ROS) generation and reduced Photosystem II (PSII) photochemistry efficiency.

The dissolution of nanoparticles leads to the production of metal ions such as Zn^2+^ and Cu^2+^ [74, 80], which quickly bind to biomolecules like proteins and carbohydrates, thereby inhibiting vital microbial processes [81]. Joe et al. [82] discovered that the antimicrobial activity of ZnO NPs in dark conditions, which excluded the effect of reactive oxygen species (ROS), was primarily attributed to the disruption of the microbial cell wall by ZnO NPs and the elevation of Zn2 + ion concentration within the microbial cytoplasm.

In our experiments, we observed that ZnO nanoparticles were more potent than CuO nanoparticles. This discrepancy could be attributed to the smaller size of ZnO nanoparticles (11.5 nm) compared to CuO nanoparticles (24.5 nm). Unlike the findings of Franklin et al*.* [83], who did not observe a size-dependent effect, numerous studies have demonstrated the significant influence of particle size on antibacterial activity.

The band gap energy of zinc oxide (ZnO) and copper oxide (CuO) nanoparticles (NPs) plays a crucial role in determining their antifungal activity. A higher band gap value has been found to be associated with increased antifungal activity, as noted by Yassin et al. [84]. In a study conducted by Amuthavalli et al. [85], ZnO NPs with a direct band gap of 3.4 eV were prepared and demonstrated both antibacterial and antifungal properties. Similarly, Roy and Rhim synthesized CuO NPs with a direct band gap of 3.4 eV, which exhibited potent antibacterial activity [86].

Overall, it appears that there is a crucial relationship between nanoparticle size and their impact on microbes, as emphasized by Yamamoto [87] and Siddiqi et al*.* [71]. From this perspective, our study highlights the effectiveness of ZnO and CuO nanoparticles as viable alternatives to phytotoxic fungicides for managing late blight disease.

In our study, we acknowledge a notable limitation related to the absence of a positive control group. While we rigorously compared the antifungal activity of ZnO and CuO NPs with a negative control, our primary objective was to evaluate the efficacy of ZnO and CuO NPs as antifungal agents. Notably, this research represents the first investigation into the effectiveness of ZnO NPs against *P. infestans*, both in vitro and in vivo. By excluding a positive control, we aimed to isolate the effects of ZnO and CuO NPs without potential interference from other antifungal substances.

The negative control served as a baseline, allowing us to assess whether ZnO and CuO NPs exhibited any antifungal activity beyond what would be expected from a non-antifungal substance. While our study design intentionally omitted a positive control, we recognize the importance of future investigations that incorporate positive controls. Such studies could provide a more comprehensive comparison and validate our findings.

## Conclusion

This study found that ZnO and CuO nanoparticles had significant antifungal activity against *P. infestans*, which causes late potato blight. Both ZnO and CuO NPs were effective both in vitro and in vivo. Consequently, these nanoparticles can be considered as a safe and eco-friendly substitute for chemical fungicides in the management of potato late blight. However, it is important to note that this study was of a short-term nature, limited to in vitro and in vivo experiments. Therefore, conducting long-term field studies alongside comparisons with currently in use fungicides and assessing the phytotoxicity of ZnO and CuO NPs on potato plants are necessary steps to validate the reliability of our findings in real-world conditions.

## Data Availability

The datasets generated and analyzed during the current study are available in the National Center of Biotechnology Information's (NCBI), The *P. infestans* isolates (Pi Alharethi YEM2021) has been uploaded successfully under the accession number GenBank OQ119020 website's, https://www.ncbi.nlm.nih.gov/nuccore/2417000057 [28].

## Abbreviations

AUDPC: Area under the disease progress curve
CuO: Copper oxide
DSI: Disease severity index
FT-IR: Fourier-transform infrared spectroscopy
HR-TEM: High-resolution transmission electron microscopy
JCPDS: Joint committee on powder diffraction standards
LSD: Least significant difference
NCBI: National center of biotechnology information's
ROS: Reactive oxygen species
SAED: Selected area electron diffraction
SPPC: Seed potato production center
SPSS: Statistical package for social sciences
TEM: Transmission electron microscopy
UV–Vis: Ultraviolet–visible
XRD: X-ray diffraction
ZnO: Zinc oxide
